# Adsorption Properties of Tetracycline onto Graphene Oxide: Equilibrium, Kinetic and Thermodynamic Studies

**DOI:** 10.1371/journal.pone.0079254

**Published:** 2013-11-26

**Authors:** Ehsan Ezzatpour Ghadim, Firouzeh Manouchehri, Gholamreza Soleimani, Hadi Hosseini, Salimeh Kimiagar, Shohreh Nafisi

**Affiliations:** 1 Young Researchers and Elite Club, Central Tehran Branch, Islamic Azad University (IAUCTB), Tehran, Iran; 2 Department of Chemistry, Islamic Azad University, Central Tehran Branch (IAUCTB), Tehran, Iran; 3 Department of Physics, Islamic Azad University, Central Tehran Branch (IAUCTB), Tehran, Iran; 4 Department of Chemistry, Shahid Beheshti University, Evin, Tehran, Iran; University of Quebect at Trois-Rivieres, Canada

## Abstract

Graphene oxide (GO) nanoparticle is a high potential effective absorbent. Tetracycline (TC) is a broad-spectrum antibiotic produced, indicated for use against many bacterial infections. In the present research, a systematic study of the adsorption and release process of tetracycline on GO was performed by varying pH, sorption time and temperature. The results of our studies showed that tetracycline strongly loads on the GO surface via π–π interaction and cation–π bonding. Investigation of TC adsorption kinetics showed that the equilibrium was reached within 15 min following the pseudo-second-order model with observed rate constants of k_2_ = 0.2742–0.5362 g/mg min (at different temperatures). The sorption data has interpreted by the Langmuir model with the maximum adsorption of 323 mg/g (298 K). The mean energy of adsorption was determined 1.83 kJ/mol (298 K) based on the Dubinin–Radushkevich (D–R) adsorption isotherm. Moreover, the thermodynamic parameters such as ΔH°, ΔS° and ΔG° values for the adsorption were estimated which indicated the endothermic and spontaneous nature of the sorption process. The electrochemistry approved an ideal reaction for the adsorption under electrodic process. Simulation of GO and TC was done by LAMMPS. Force studies in z direction showed that tetracycline comes close to GO sheet by C_8_ direction. Then it goes far and turns and again comes close from amine group to the GO sheet.

## Introduction

**Figure 1:**
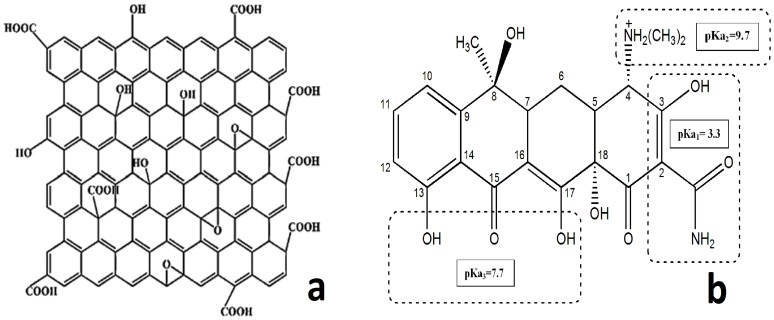
Structure of graphene oxide (a), Structure of tetracycline and pKa values (b).

Graphene oxide (GO) (Fig. 1a), a derivative of graphene, is a non-toxic and more biocompatible than other existing nanoparticles, such as quantum dots, noble metals (gold/silver nano-particles), and rare-earth ions (upconversion nanocrystals) [1]. It is a sp^2^-bonded carbon sheets with individual physical and chemical properties which has attracted remarkable attention since 2004 [2]. Many researches have explored the potential of GO for variety biomedical applications such as electrochemical devices [3], [4], energy storage [5], [6], catalysis [7], adsorption of enzyme, cell imaging and drug delivery, as well as biosensors. Most of the antibiotic drugs like TC have appropriate interaction with GO via π-π stacking [8], [9]. Large quantities of oxygen atoms in the forms of epoxy, hydroxyl, carboxyl groups and delocalized conjugated π electrons on the surface of the GO [5] builds it extremely hydrophilic and provides the capability to apply GO in the aquatic and biological environment.

Tetracycline (TC, C_22_H_24_N_2_O_8_) (Fig. 1b) is the second greatest antibiotic which is extensively used in the world. It exhibits broad-spectrum antimicrobial activity against a variety of diseases. Tetracyclines are pluripotent drugs that inhibit the activity of matrix metalloproteinases (MMPs) and affect many cellular functions including proliferation, migration, and matrix remodeling [10]. TC has a planar structure consisting of four fused rings with hydrophilic groups on one face, hydrophobic groups on the other face and each ring including phenol, alcohol, ketone and amino.

Controlling delivery systems are used to improve therapeutic efficacy and safety of drugs by delivering them at a rate dictated which is needed due to the physiological environment during a period of treatment to the site of action [11]. Many problems are minimized when the drug release process is slow [12]. New drug delivery vehicles such as liposomes, dendrimers and graphene oxide nanoparticles offer a promising way to improve bioavailability, efficacy and specificity of pharmaceutical compounds in general [13].

In the present work, GO was prepared and used as an adsorbent to deal with tetracycline at different pHs, sorption times and concentrations. Free Gibbs energy, enthalpy, entropy, and activation energy due to pseudo first and second order equations were calculated. The adsorption kinetics, isotherms and thermodynamics of TC on GO were systematically approved the π-π interaction mechanism between them. The mechanism for adsorption of tetracycline on GO was deduced from fitting adsorption isotherms. In order to study the behavior of TC on GO surface, simulation of GO and TC was done by LAMMPS.

## Materials and Methods

### Materials

Tetracycline hydrochloride, graphite flakes (150 µm flakes) were purchased from Sigma-Aldrich chemical Co. USA. GO nanoparticles were manufactured by improved Hummers method [14]. Other chemicals used were of analytical reagent grade and used without further purification.

### FTIR Spectroscopy

Infrared spectra were recorded on a FTIR spectrometer (100 N model), equipped with deuterated triglycine sulphate (DTGS) detector and KBr beam splitter, using AgBr windows. Solution of TC (4 g/L) was added dropwise to the GO (4 g/L) solution and mixed thoroughly by a vortex mixer. The suspension was incubated at 25°C overnight and wrapped in aluminum foil to avoid possible photodegradation of tetracycline. The suspension was centrifuged at 12000 rpm for 15 min. The supernatant was collected for FTIR measurements using hydrated films [15]. Interferograms were accumulated over the spectral range 4000–600 cm^−1^ with a nominal resolution of 4 cm^−1^ and 100 scans.

### UV-Visible Spectroscopy

The UV-Vis spectra were recorded on a Perkin-Elmer Lambda spectrophotometer with a slit of 2 nm and scan speed of 400 nm/min. Quartz cuvettes of 1 cm were used. The absorbance assessments were performed at pH 7.0 by concentration of GO (20 mg/L), TC (100 mg/L) and GO-TC.

### Voltammetric Experiments

Voltammetric experiments were performed using a µAutolab Type III electrochemical system. A conventional three-electrode cell consisting of a glassy carbon working electrode (modified and unmodified), a platinum wire counter electrode and a saturated Ag/AgCl reference electrode were used. Glassy carbon electrode (GCE) was cleaned by polishing with 0.05 µm alumina slurry on a polishing cloth to create a mirror finish; the electrode was then rinsed thoroughly with double-distilled water and then dried under ambient temperature. Typically, a stable suspension of graphene oxide containing 2.0 mg/ml in DMF using 30 min ultrasonic agitation was prepared. After the electrode surface was air dried, 5.0 µL of this suspension was cast onto the surface of the pretreated GC electrode by a micro syringe and then it was dried in air.

### General Adsorption Experiments

To demonstrate the release efficiency directly, the adsorption experiments were performed using a series of 50 mL flasks containing 20 mg/L GO and 25 mL (6–180 mg/L) TC solutions. The pH of the solutions were adjusted to 6–7 by adding HCl or NaOH. The mixtures of GO and TC solutions were incubated overnight and used for determination by UV–Vis absorbance at 356 nm.

To deliberation the influence of pH on adsorption, the mixtures of GO (2 ml of 20 mg/L) and TC solutions (4 ml of 10–100 mg/L) were prepared. The pH of solutions were adjusted from 2 to 10 using NaOH and HCl and incubated overnight. The supernatant was collected for determination by UV–Vis absorbance at 356 nm. The adsorption percentage (Ads. %) was calculated based on the equation (1) :
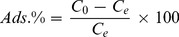
(1)


C_0_ and C_e_ are the initial and the equilibrium concentrations of TC in solution phase, respectively.

To investigate the kinetics of the adsorption, 2 mL GO (20 mg/L) was mixed with 4 mL of different concentrations of tetracycline (6–180 mg/L) (pH = 3.6). The mixtures were taken for centrifugation with 15 min interval after vigorous mixing by vortex mixture. On regular time intervals, the concentrations of tetracycline in supernatant were determined successively by UV–Vis absorbance at 356 nm. The rate constants were calculated using the conventional rate expression.

The effect of temperature on the sorption of tetracycline by GO was estimated by incubating the samples (2 ml GO 20 mg/L) and 4 mL of different concentrations of tetracycline (6–180 mg/L) overnight and then beneath different temperatures (298, 308, and 318 K). The temperature was preserved through the procedure of investigation including under centrifugation. The supernatant was collected for determination by UV–Vis absorbance at 356 nm.

Adsorption isotherm studies were carried out with constant concentration of GO (20 mg/L) and different concentrations of tetracycline solutions (6–180 mg/L), pH = 3.6 and different temperatures; 298, 303 and 308 K [16]. GO solutions were incubated with tetracycline overnight and covered by aluminum foil to refraining probable photo degradation of tetracycline [17]. Then suspension was centrifuged at 6000 rpm for 30 min. The supernatant was gathered for determination by UV–Vis absorbance at 356 nm, drawing a calibration curve manufactured with tetracycline solutions of different concentrations. The absorption experiments were performed under the same condition, but different temperatures (298, 303 and 308 K).

### Simulation

LAMMPS (Large-scale Atomic/Molecular Massively Parallel Simulator) is a classical molecular dynamics code that models an ensemble of particles in a liquid, solid, or gaseous state. It can model atomic, polymeric, biological, metallic, granular, and coarse-grained systems using a variety of force fields and boundary conditions. In the most general sense, LAMMPS integrates Newton’s equations of motion for collections of atoms, molecules, or macroscopic particles that interact via short- or long-range forces with a variety of initial and/or boundary conditions. For computational efficiency, LAMMPS uses neighbor lists to keep track of nearby particles. The lists are optimized for systems with particles that are repulsive at short distances, so that the local density of particles never becomes too large.

In the present research, we used adaptive intermolecular reactive bond order (AIREBO) potential using numerical. Both the repulsive and attractive pair interaction functions are modified to fit bond properties. Long range atomic interactions and single bond torsional interactions are also included [18], [19].

## Results

### Adsorption of Tetracyclines by Graphene Oxide Suspension

FT-IR and UV–Vis spectroscopy were employed to investigate the adsorption of tetracycline on GO.

### FTIR Spectral Analysis of Tetracycline-graphene Oxide

**Figure 2:**
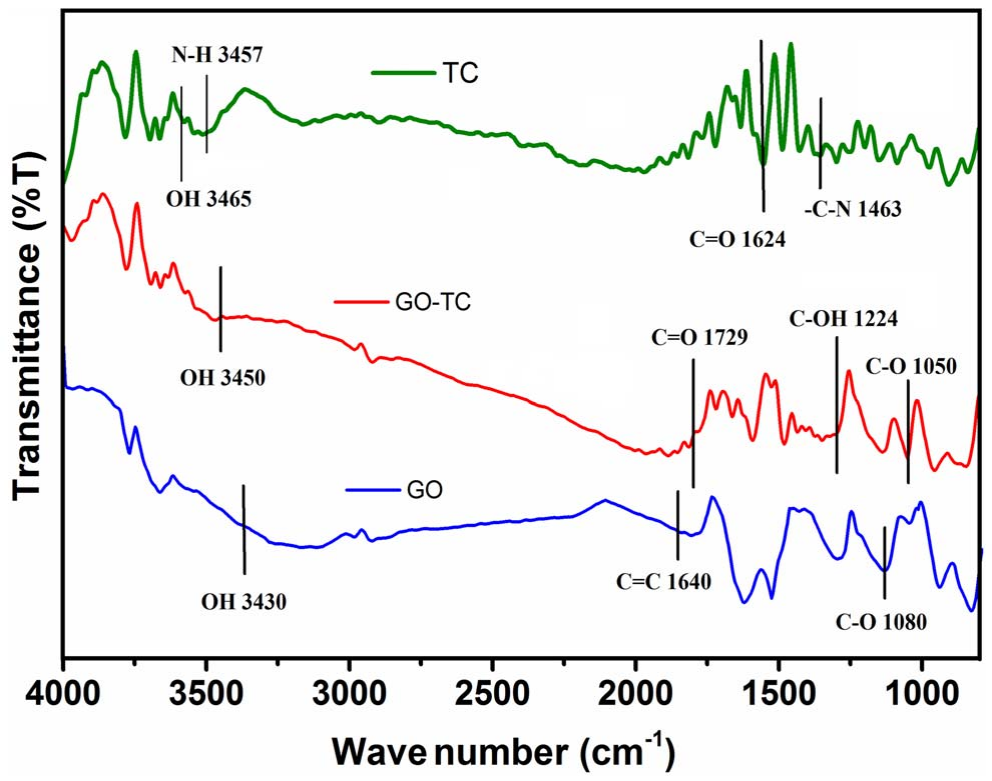
FTIR spectra of free TC, free GO and TC after adsorption on GO (GO-TC).

Transform Infrared (FTIR) spectra of TC showed apparent characteristic bands at 3465 cm^−1^ (O–H), 3457, 3441 cm^−1^ (N–H), 1624 cm^−1^ (C = O) and 1463 cm^−1^ ( = C–N) (Fig. 2). In GO-TC complex, shifting of the TC absorption bands to 3450 cm^−1^ (O-H), 1729 cm^−1^ (C = O), and appearance of the new bands at 1224 cm^−1^ (C-OH), and 1050 cm^−1^ (C-O), suggesting that oxygen-containing groups are introduced into the graphene structure (Fig. 2). Go and TC stick to each other and form bundles, and the space between the bundles provides more adsorption sites [20]. Water molecules could form H-bands with functional groups on GO, which blocks the access of TC molecules into the sorption sites.

### UV-Visible Spectra

**Figure 3:**
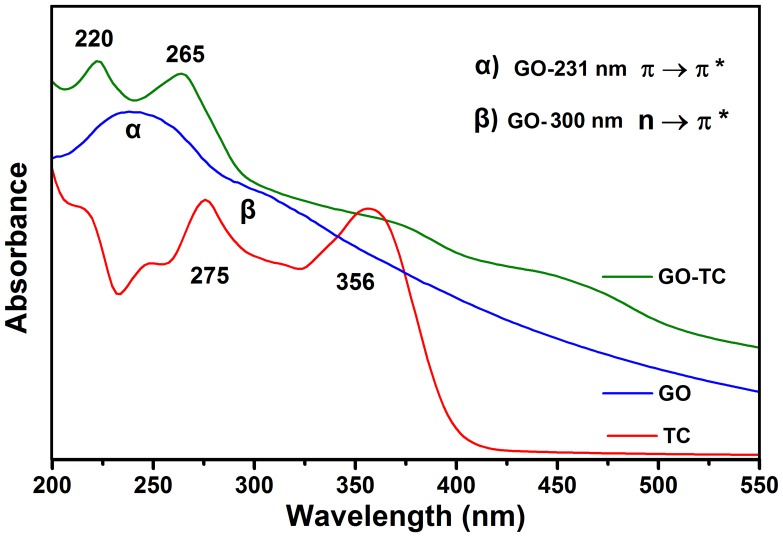
The UV–Vis absorption spectra of free TC, free GO and TC after adsorption on GO (GO-TC).

The absorption spectra of GO, tetracycline, and GO–TC are shown in Fig. 3. The GO dispersion displays a maximum absorption at 231 nm, which is due to the π–π* transition of aromatic C = C bands (Fig. 3). Furthermore, a similar shoulder band around ∼300 nm is observed which can be attributed to n→π* transitions of the carbonyl groups [21]. The TC absorption bands are located at 356 and 275 nm which blue shifted and appeared at 265 and 220 nm upon adsorption on GO. The adsorption process is possibly the non-electrostatic–dispersion interaction between bulk systems on GO surface and TC molecules contained both benzene rings and double bands (C = C, C = O), or hydrophobic and π–π electron donor–accepter interaction between GO and TC. The cation–π bonding may happen between the easily protonated amino group which is on the ring C_4_ of the tetracycline molecule and the graphene π-electrons. Similar interactions are observed in graphite and carbon nanotubes [9], [22].

### Effect of pH

In the present research, different concentrations of TC (6.0–180.0 mg/L) were applied to examine the sorption behavior of TC on a constant concentration of GO (2 ml of 20 mg/L) at different pH values (2–10 with an interval 1 unit) using UV-Vis spectroscopy. The adsorption percentage (Ads. %) was calculated based on the equation (1).

**Figure 4:**
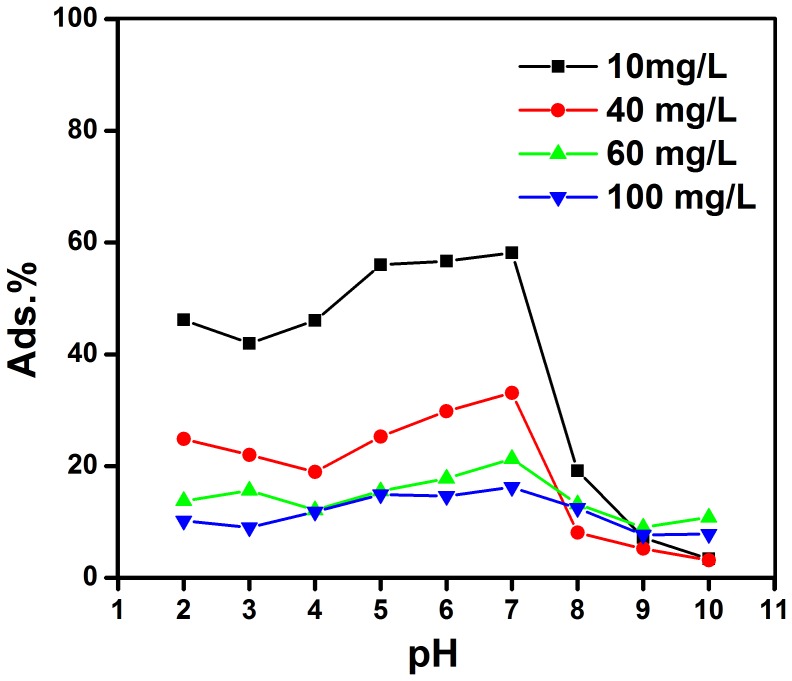
Effect of pH on the adsorption efficiency of TC (10.0–100.0 mg/L) on GO (20.0 mg/L); Temp. 25±0.1.

It should be noted that tetracycline has variable charges on different sites depending on solution pH. When pH is under 4, TC exists as a cation (TCH^3+^), due to the protonation of dimethyl-ammonium group. At pH between 3.5 and 7.5, TC exists as a zwitterion (TCH_2_^0^), due to the loss of a proton from the phenolic diketone moiety. At pH upper than 7, TC exists as anion (TCH^−^ or TC^2−^) due to the loss of protons from the tri-carbonyl system and phenolic di-ketone moiety [23]. By increasing the pH from 4 to 7, the adsorption of TC on GO increases (Fig. 4). The maximum adsorption occurs at pH 7. At pH higher than 7, the adsorption percentages decreases. For concentrations less than 40 mg/L, the adsorption percentage was found to be above 50% (pH 5–7). The variation in pH can not only focuses on the protonation–deprotonating transition of functional groups on GO, but also results in a change in chemical speciation for ionizable organic compounds. The above results are comparable with biological systems in which the pH inside the cell is 5 and out of cell is 7. This phenomena sufficiently approves that pH affects absorption of TC on GO and can be used to predict the TC release from GO inside the cells. The above results show that GO can act as an appropriate carrier for drug delivery systems.

### Adsorption Kinetics

In order to investigate the adsorption process of TC on GO, pseudo-first-order and pseudo-second-order kinetics model were used.

**Figure 5:**
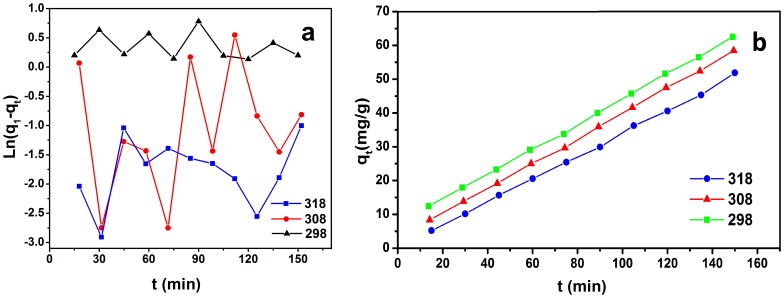
The pseudo-first-order (a) and the pseudo-second-order (b) kinetics model for adsorption of tetracycline on GO suspension (20.0 mg/L), pH = 3.6, T = 298, 308, 318 K.

Pseudo-first-order model:

The pseudo-first-order equation is given as Eq. (2) [16], [20], [24]:
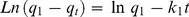
(2)Where q_1_ and q_t_ are the amount of TC adsorbed on the sorbent (mg/g) at equilibrium and at time t, respectively, and k_1_ is the rate constant of the first-order adsorption (min^−1^). The values k_1_ for TC adsorption on GO were defined from the plot of Ln (q_1_ − q_t_) against t (Fig. 5a).

Pseudo-second-order model:

The pseudo-second-order model is represented as Eq. (3) [25]:
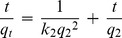
(3)Where k_2_ is the rate constant of the second-order adsorption (g/mg min). The straight-line plots of q_t_ versus t (Fig. 5b) have been tested to obtain rate parameters. The second-order rate constants were used to calculate the initial sorption rate h (mg/g min) [26], given by:



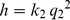
(4)The batch kinetic data based on our investigation were appropriately satisfying the condition of second-pseudo order models. In the present study, Ho’s pseudo-second-order kinetics model was exploited to examine the fitness of the experimental data and to evaluate the kinetics of the adsorption of tetracycline on GO. The pseudo-second-order kinetics model was based on the hypothesis that the rate-limiting step includes chemisorption, which has been extensively applied to the sorption of contaminants from aqueous solutions in recent years [27]. So, only second-pseudo order models effectively describe the kinetic data at 95% confidence level. The consequences of the kinetic parameters and the calculated initial sorption rate values are recorded in Table 1. Based on the correlation coefficients, the adsorption of TC is finest described by the pseudo-second-order model. In an assumed adsorption system, the initial adsorption rate intensified by increasing the temperature. Furthermore, it was probable to calculate the activation energy (E_a_) for the adsorption employing the Arrhenius equation [28] based on the k values.
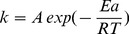
(5)Where A is the frequency factor (min^−1^), E_a_ is the activation energy (kJ/mol), R is the ideal gas constant (kJ/mol K), and T is the temperature (K) [29].

**Table 1:** Kinetic parameters for TC adsorption on GO at different temperatures.

	Pseudo-second-order model			
T (K)	k_2_ (1/min)	q^2^ (mg/g)	h (mg/g min)	r^2^
**298**	0.3468	198.54	68.89	0.996
**308**	0.2742	381.77	104.7	0.994
**318**	0.5362	411.76	221.8	0.986

Eq. (5) can be converted into Eq. (6) by taking logarithm:
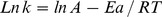
(6)


Thus, E_a_ could be obtained from the slope of the line plotting ln k versus 1000/T, the estimated E_a_ for TC adsorption on GO was 3.2411 kJ/mol. The lower the E_a_ was, the fewer sensitive the temperature effected on the adsorption reaction. It has been proved that the process can be simply conducted.

### Adsorption Isotherms

**Figure 6:**
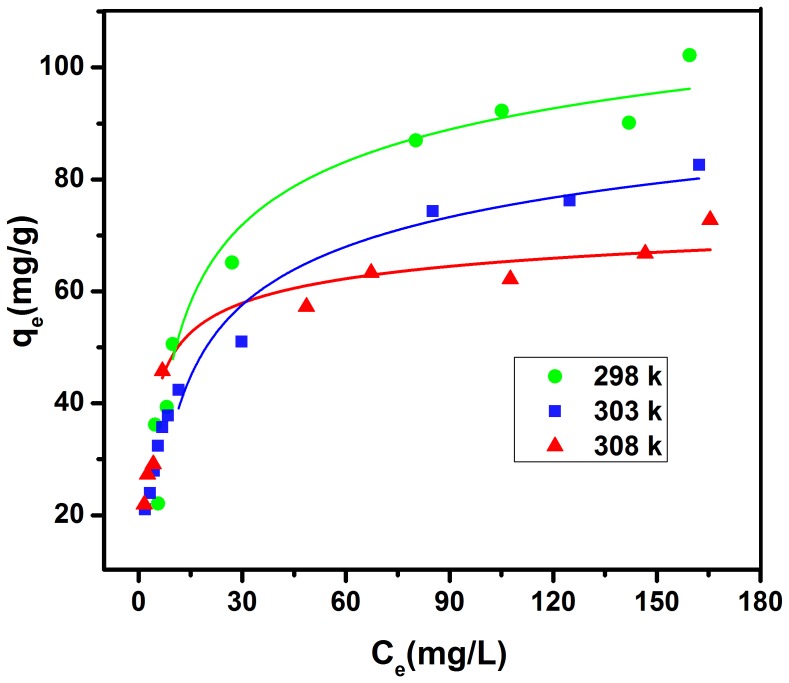
Isotherm of TC (6.0–180.0 mg/L) adsorption on GO (20.0 mg/L) at different temperatures (298, 303, 308 K).

Adsorption isotherms describe how solutes interact with sorbents. Adsorption isotherms and the equilibrium adsorption amount of TC on GO as a function of equilibrium concentration of TC is depicted in Fig. 6. The absorption data were fitted to both Langmuir and Freundlich model which are often described by equilibrium sorption isotherms model [30]:
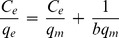
(7)Where C_e_ is the equilibrium concentration of TC (mg/L), q_m_ is the maximum monolayer adsorption (mg/g), q_e_ is the amount of TC adsorbed per unit weight of GO at equilibrium concentration (mg/g) and b is the Langmuir constant related to the affinity of binding sites (L/mg). The equilibrium concentration (C_e_) of TC was calculated mentioning to the calibration curve of TC [31]. Moreover, the widely used empirical Freundlich equation basis on sorption on a heterogeneous surface is given by [32]:
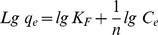
(8)where KF and n are Freundlich constants indicating the sorption capacity (mg/g) and intensity, respectively. The Langmuir-Freundlich isotherm constants were determined from the plots of Ce/qe against Ce, lg qe versus lg Ce, respectively, at 298, 303, 308, 310 and 313 K. The isothermal constants and the correlation coefficients are depicted in Table 2. Langmuir and Freundlich isotherm models were statistically important at a 95% confidence level. It is found that the adsorption of TC on GO correlated well (r>0.99) with the Langmuir equation as compared to the Freundlich equation (r>0.95) underneath the studied concentration range. Therefore, the Langmuir isotherm fits appropriate compared with the Freundlich isotherm in all conditions according to the correlation coefficients r. The maximum adsorption capacity of TC on GO was 322.43, 101.87, 73.53 mg/g at 298, 303, 308 respectively. The shape of the isotherm has been discussed in order to predict whether an adsorption system is desirable or undesirable. The vital property of the Langmuir isotherms can be expressed by means of ‘RL’, a dimensionless constant related to the separation factor or equilibrium parameter. RL is computed using the following equation [25], [31]:

**Table 2:** Langmuir, Freundlich and D–R constants and correlation coefficients of TC adsorption on GO at different temperatures.

	Langmuir			Freundlich			D–R*		
T (K)	q_m_(mg/g)	R_L_	r^2^	K_F_ (mg/g)	n	r^2^	q_m_(mg/g)	E(kj/mol)	r^2^
**298**	322.43±1.25	0.01787	0.997	21.40	3.656	0.943	24.62±0.74	1.83	0.961
**303**	101.87±1.08	0.00846	0.993	20.09	3.762	0.962	22.92±0.59	1.69	0.988
**308**	73.53±0.55	0.2546	0.998	20.13	3.721	0.983	24.94±0.61	1.40	0.988

* Dubinin–Radushkevich.



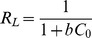
(9)Where, C_0_ is the initial TC concentration (mg/L) and b is the Langmuir adsorption of equilibrium constant (L/mg). The calculated R_L_ values are recorded in Table 2. In the present investigation, the equilibrium parameter R_L_ was found to be between 0 and 1, hence the sorption process was quite favorable and the adsorbent employed demonstrated a good potential for the sorption of TC. Finally, the Dubinin–Radushkevich (D–R) isotherm was also examined in its linearized form:
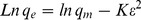
(10)Where q_e_ and q_m_ have the same meaning as above, K is the parameter linked to the adsorption energy. ε is the adsorption potential, explained the Polanyi as the free energy change needed to transfer a molecule from bulk solution to the adsorption region. The Polanyi potential differences with the concentration according to [33]:
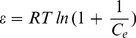
(11)where R is the ideal gas constant and T is the temperature (K). A linear correlation is manufactured by plotting ln qe versus ε2 (shown in Table 2), indicating that TC adsorption also obeys the D–R equation. The adsorption energy for TC adsorption can be determined by:



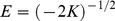
(12)The values of the adsorption energy were estimated as 1.83, 1.69 and 1.4 kJ/mol, at 298, 303, 308 K respectively, indicating that the values lie within the energy range of physical adsorption, i.e., <8 kJ/mol.

### Thermodynamic Studies

The sorption manners of different concentrations of TC onto GO were critically explored at 298, 303, 308, 310 and 313 K, respectively. Thermodynamic parameters were computed from following equations:
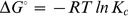
(13)where R is the universal gas constant (8.314 kJ/mol K), T is the temperature (K) and K_c_ is the distribution coefficient. Gibbs free energy change of adsorption (ΔG°) was calculated using ln K_c_ values for different temperatures. The K_c_ value was calculated using following equation [34]:
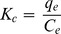
(14)where Ce is the equilibrium concentration of TC and qe is the amount of TC adsorbed per unit weight of GO at equilibrium concentration (mg/g). The enthalpy change (ΔH°) and entropy change (ΔS°) of adsorption were estimated from the following equation [35]:



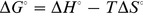
(15)According to Eq. 15, ΔH° and ΔS° factors can be calculated from the slope and intercept of the plot of ΔG° against T, respectively. The thermodynamic parameters were shortened in Table 3. The positive values of ΔH° and the negative values of ΔG° show the endothermic and spontaneous nature of sorption process.

**Table 3:** Thermodynamic parameters for the adsorption of TC on GO.

			ΔG° (kJ/mol)		
C_0_(mg/L)	ΔH°(kJ/mol)	ΔS°(kJ/mol K)	298 K	303 K	308 K
20.0	44.045	0.2553	−2.0068	−3.2704	−2.1731

### Electrochemical Studies

**Figure 7:**
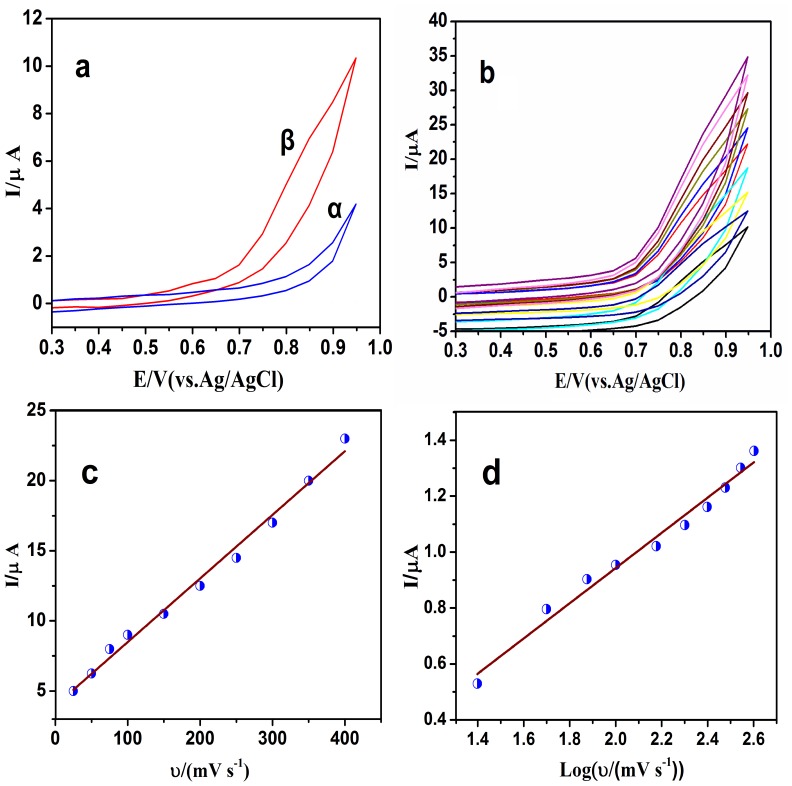
CV curve of tetracycline (1 mM, in phosphate buffer solution, 0.1 M, pH = 7) at 50 mV/s (a); CV curve of tetracycline (1 mM) at different scan rates: (from Bottom to up) 25, 50, 75, 100, 150, 200, 250, 300, 350, 400 mV/s (b); Observed dependence of peak current on the scan rate (c); Plot of variation of peak current with the logarithm of scan rate (d).

The voltammetric response of graphene oxide/glassy carbon electrode (GO/GCE) in the absence and presence of tetracycline (1.0 mM in phosphate buffer, 0.1 M, pH 7.0, scan rate of 50.0 mV/s) is shown in Fig. 7. The cyclic voltammetric behavior of the tetracycline evidenced one broad oxidation peak with the peak potential at Epa = 0.84 V during the anodic scan (Fig. 7a). No peaks were observed in the reverse scan, suggesting the irreversible nature of the oxidation process of tetracycline at the GO/GCE.

In the electrochemical investigations, useful information involving the electrochemical reaction mechanisms usually can be obtained from the potential scan rate. Therefore, the electrochemical behavior of tetracycline (1.0 mM) was investigated at pH 7.0 and scan rate from 25 to 400 mV/s by cyclic voltammetry (Fig. 7b). As shown in Fig. 7c, by increasing the scan rate from 25 to 400 mV/s, a linear relationship was observed between the peak intensity Ipa and the scan rate υ (Fig. 7b), indicating that the oxidation of tetracycline at GO/GCE is an adsorption-controlled process. The effect of scan rate on peak current was also studied under the above conditions with a plot of log I (logarithm of peak current) vs. log υ (logarithm of scan rate), giving a straight line with a slope of 0.91 (Fig. 7d). This value is close to the theoretical value of 1, which is expected for an ideal reaction for the adsorption controlled electrodic process [36].

### Simulation

**Figure 8:**
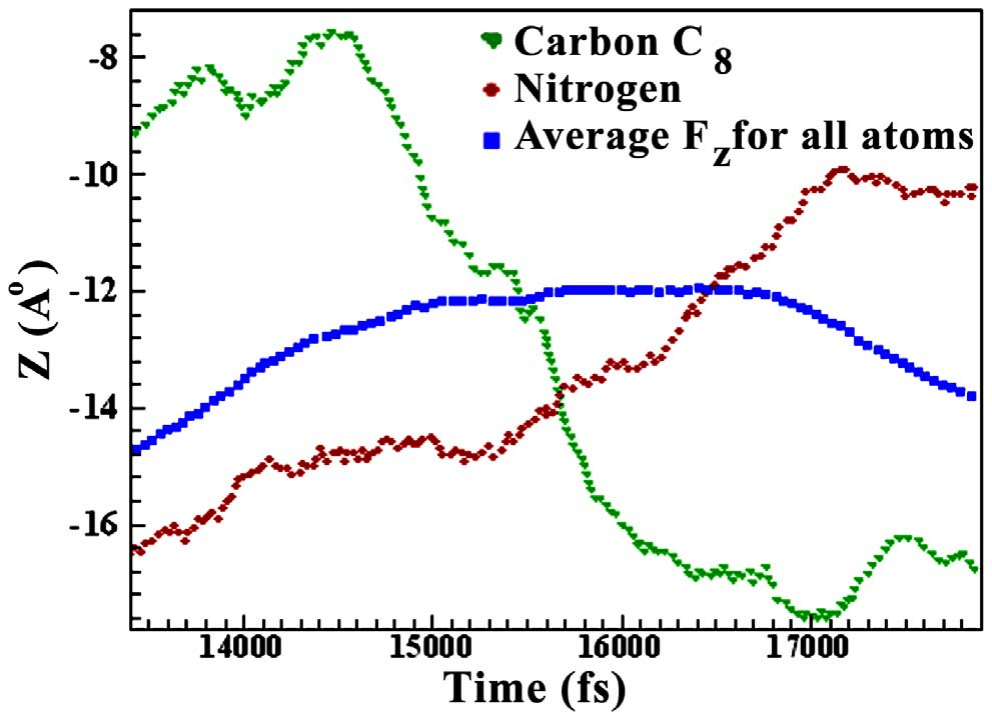
Variation of z *vs*. time for C_8_ (triangle symbol) and amino (circle symbol). Square symbol is z average over all TC molecules.

The behavior of TC near the GO sheet was studied by Lammps code. GO was considered as 25×25 nanometer square sheet and z = −5 position with periodic boundary condition using the Adaptive Intermolecular Reactive Empirical Bond Order (AIREBO) potential. The AIREBO model is a function for calculating the potential energy of covalent bonds and the interatomic force. In this model, the total potential energy of system is a sum of nearest-neighbor pair interactions which depends not only on the distance between atoms but also on their local atomic environment. A parameterized bond order function was used to describe chemical pair bonded interactions. The adaptive intermolecular reactive bond order (AIREBO) potential, in which both the repulsive and attractive pair interaction functions are modified to fit bond properties, and the long-range atomic interactions and single bond torsional interactions are included [18]. The AIREBO model has been used in recent studies using numerical [19]. Center of TC is at the zero point of coordinate and force cut of radius is 10 Å. Temperature increases to 25°C after 1000 run step and steady state has been considered after 2×10^5^ run step. It has been averaged over z. Figure 8 shows variation of z *vs.* time for C_8_ (triangle symbol) and amino (circle symbol). It can be seen that the distance between TC (from the C_8_ side) and GO sheet decreases to 14400 fs. Then TC turns and goes close to GO sheet from the amino side. In this case the center of TC is 7 Å far from GO sheet. The closest distances for the C_8_ and amino side are 2.6 and 5 Å respectively. The curve with square symbol is z average across TC molecules (Fig. 8).

**Figure 9:**
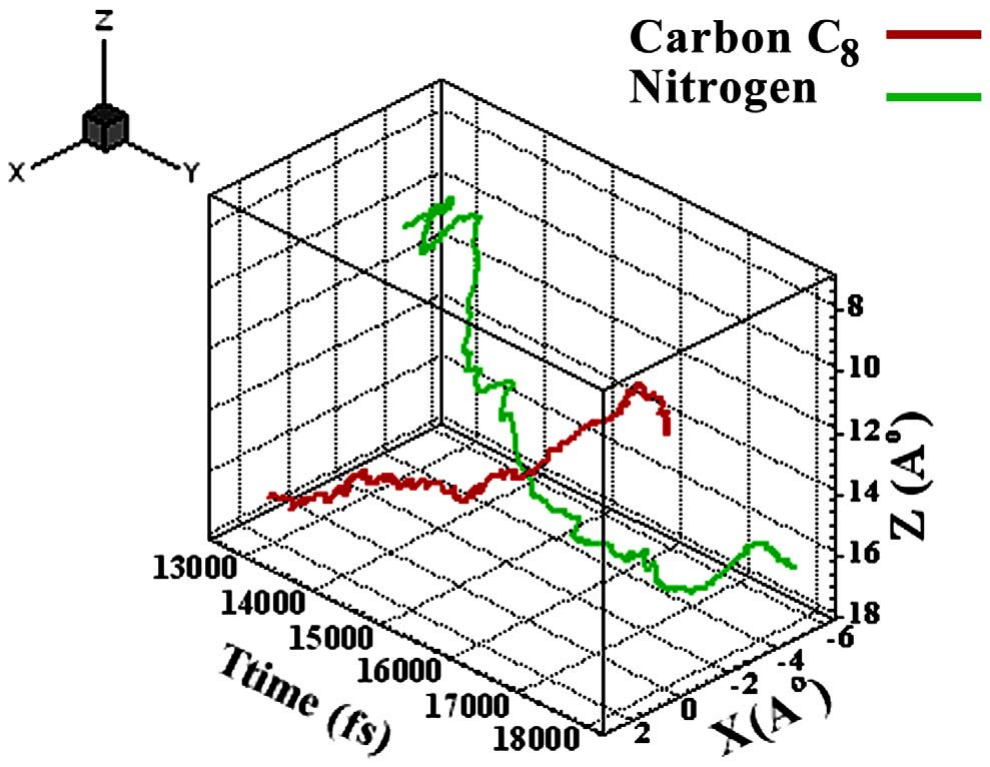
Variation of z and x *vs*. time.

**Figure 10:**
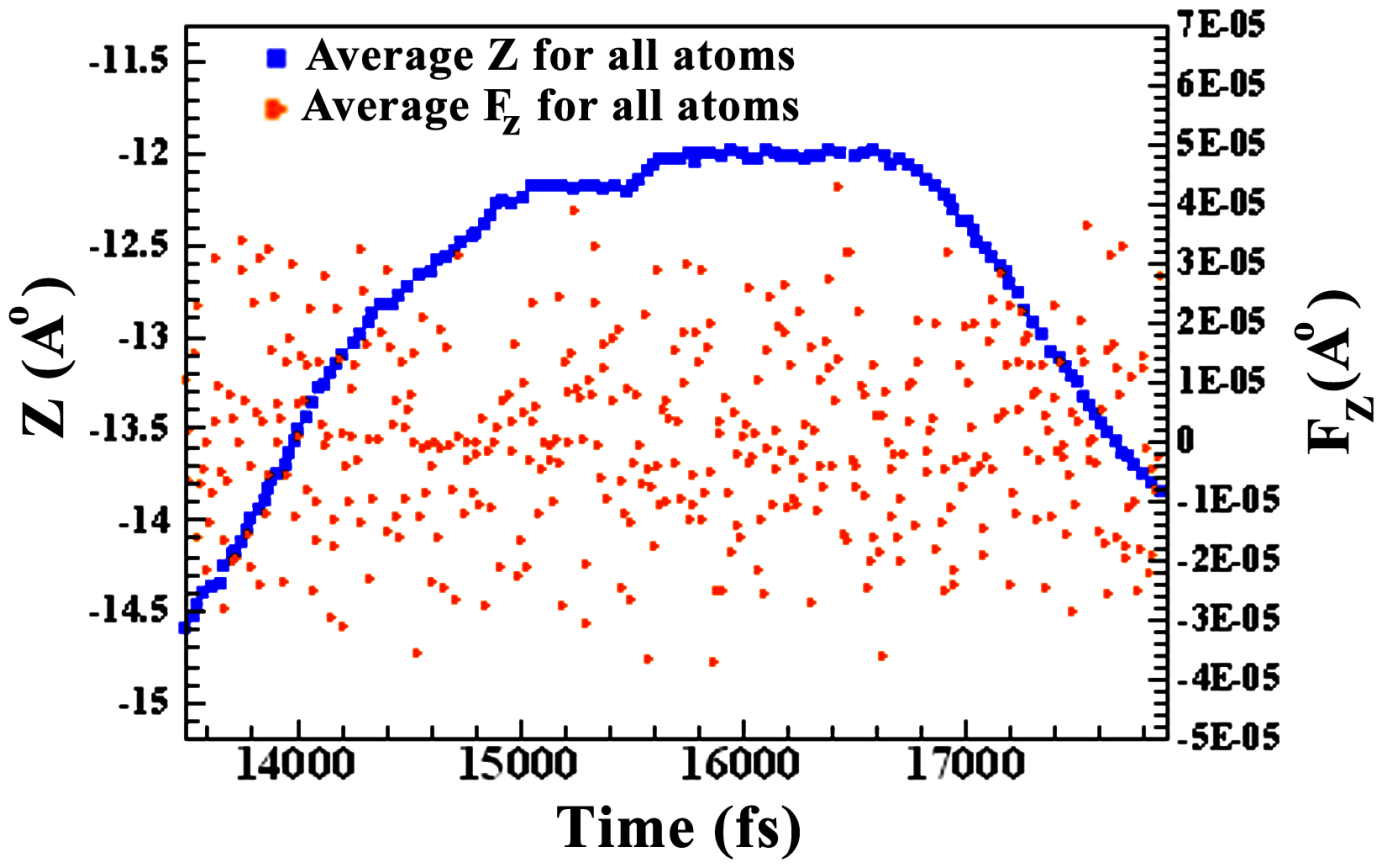
Variation of resultant force in the z direction *vs.* time.

Variation of x and z *vs*. time has been shown in Fig. 9. 3D curves show a clear turning of TC. The resultant force in the z direction changes with time (Fig. 10). The triangle symbol implies absorption and repulsion force towards GO. Molecules with absorption force less than 15000 fs, and repulsion force greater than 18000 fs are dominated which means that TC goes close to and far from GO sheet, and turns at time 15000–17000 fs since two forces are equal. These results are in agreement with Fig. 8. The simulation results are consistent with FTIR and UV results which approved the complex formation between GO and TC.

## Conclusion

The adsorption behavior results show high adsorption efficiency of TC on GO. The adsorption mechanism is non-electrostatic dispersion and hydrophobic interaction between TC and GO. The ring structure in tetracycline molecule and the surface of the graphene oxide facilitate π–π interaction between them. Cation–π bonding was likely to happen between the amino group on the ring C_4_ of tetracycline and GO π-electron-rich structures. GO can effectively release TC in aqueous solution in wide range of pH from 2 to 10. The percentage of TC release by GO can reach 99.8%. Kinetic studies suggest that the equilibrium is achieved within only 15 min and the pseudo-second-order model is followed. The adsorption isotherms could be well fitted by the Langmuir adsorption isotherm equations with the maximum adsorption capacity of 322.43 mg/g (298 K) of TC on GO. The thermodynamic parameters imply that the adsorption is a spontaneous, endothermic and physisorption process.

Simulation studies show the presence of π–π stacking interactions between tetracycline and graphene’s surface. The closest distance between TC and GO is 6.2 Å. After 15000 fs and where the distance between center of TC and GO sheet is 7 Å, TC starts to turn. After 17000 fs, the amine group side of TC is moving far from graphene surface to 5 Å distance. Simulation results show that at any moment, some of the TC molecules are adsorbed and some of them are repulsed by graphene sheet which represent the equilibrium concentration (C_e_). TC molecules are in the mode of adsorption and desorption from graphene surface and the total energy of adsorption and desorption is equal to −6800 J.
